# Supplementary material for: Psychometric properties of the Chinese version of the Perinatal Bereavement Care Confidence Scale (C-PBCCS) in nursing practice
Source: PLoS One. 2022 Jan 21;17(1):e0262965. doi: 10.1371/journal.pone.0262965 (PMC8782403; doi:10.1371/journal.pone.0262965)
Supplement: S1 File — (DOC) [file pone.0262965.s001.doc]

**Supplementary file 1 The item-total correlations of all items**

| **Items** | **Item-total correlations** | **Items** | **Item-total correlations** |
| --- | --- | --- | --- |
| **Perinatal bereavement support knowledge** | |  |  |
| a1 | 0.266** | b8 | 0.646** |
| a2 | 0.423** | b9 | 0.625** |
| a3 | 0.570** | **Self-awareness** | |
| a4 | 0.753** | c1 | 0.687** |
| a5 | 0.632** | c2 | 0.647** |
| a6 | 0.460** | c3 | 0.731** |
| a7 | 0.444** | c4 | 0.563** |
| a8 | 0.558** | c5 | 0.703** |
| a9 | 0.507** | c6 | 0.737** |
| a10 | 0.664** | c7 | 0.742** |
| a11 | 0.453** | c8 | 0.678** |
| a12 | 0.650** | **Organizational support** | |
| a13 | 0.696** | d1 | 0.808** |
| a14 | 0.354** | d2 | 0.737** |
| a15 | 0.417** | d3 | 0.657** |
| **Perinatal bereavement support skills** | | d4 | 0.715** |
| b1 | 0.785** | d5 | 0.776** |
| b2 | 0.547** | d6 | 0.477** |
| b3 | 0.764** | d7 | 0.760** |
| b4 | 0.802** | d8 | 0.802** |
| b5 | 0.361** | d9 | 0.738** |
| b6 | 0.593** | d10 | 0.712** |
| b7 | 0.734** | d11 | 0.479** |
