# Supplementary material for: Psychometric properties of the Chinese version of the Perinatal Bereavement Care Confidence Scale (C-PBCCS) in nursing practice
Source: PLoS One. 2022 Jan 21;17(1):e0262965. doi: 10.1371/journal.pone.0262965 (PMC8782403; doi:10.1371/journal.pone.0262965)
Supplement: S2 File — (DOC) [file pone.0262965.s002.doc]

**Supplementary file 2 The criterial value（CR）of all items**

| **Items** | **CR** | **Items** | **CR** |
| --- | --- | --- | --- |
| **Perinatal bereavement support knowledge** | | b8 | 21.83** |
| a1 | 5.85** | b9 | 17.12** |
| a2 | 9.20** | **Self-awareness** | |
| a3 | 14.11** | c1 | 21.29** |
| a4 | 22.79** | c2 | 17.52** |
| a5 | 17.31** | c3 | 21.26** |
| a6 | 10.45** | c4 | 13.81** |
| a7 | 10.40** | c5 | 18.76** |
| a8 | 14.26** | c6 | 21.87** |
| a9 | 11.21** | c7 | 24.13** |
| a10 | 18.96** | c8 | 18.54** |
| a11 | 11.70** | **Organizational support** | |
| a12 | 19.05** | d1 | 26.89** |
| a13 | 20.61** | d2 | 20.90** |
| a14 | 7.87** | d3 | 16.62** |
| a15 | 9.25** | d4 | 19.90** |
| **Perinatal bereavement support skills** | | d5 | 23.71** |
| b1 | 25.02** | d6 | 10.02** |
| b2 | 14.20** | d7 | 22.31** |
| b3 | 23.48** | d8 | 25.84** |
| b4 | 27.11** | d9 | 20.76** |
| b5 | 6.56** | d10 | 19.99** |
| b6 | 13.40** | d11 | 10.02** |
| b7 | 21.66** |  |  |
