# Supplementary material for: Psychometric properties of the Chinese version of the Perinatal Bereavement Care Confidence Scale (C-PBCCS) in nursing practice
Source: PLoS One. 2022 Jan 21;17(1):e0262965. doi: 10.1371/journal.pone.0262965 (PMC8782403; doi:10.1371/journal.pone.0262965)
Supplement: S3 File — (DOC) [file pone.0262965.s003.doc]

**Supplementary file 3 Scree plot of the C-PBCCS**


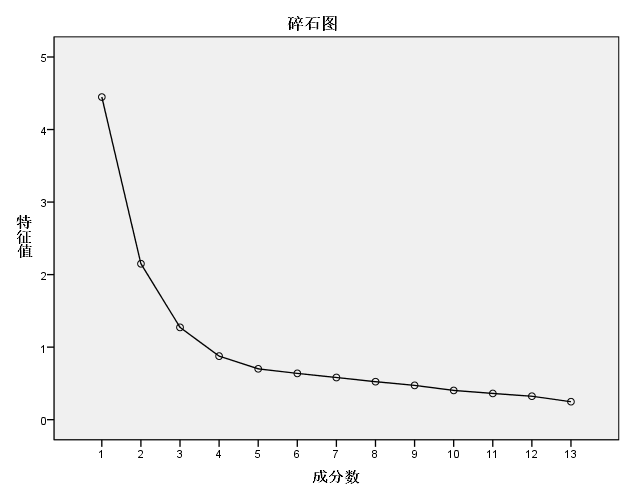


**Figure S1 Scree plot of the bereavement support knowledge scale**


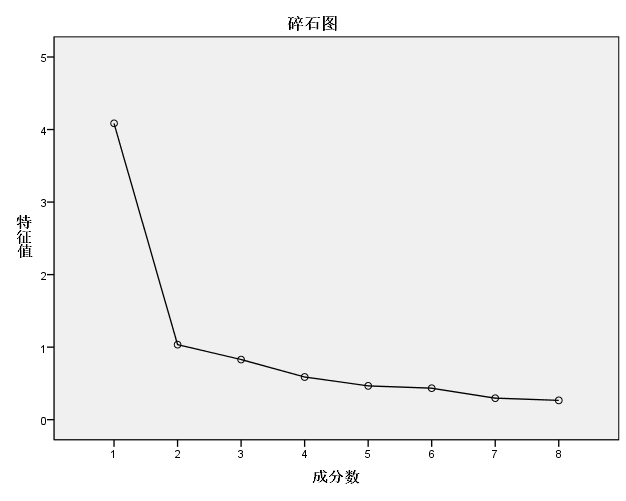


**Figure S2 Scree plot of the bereavement support skills scale**


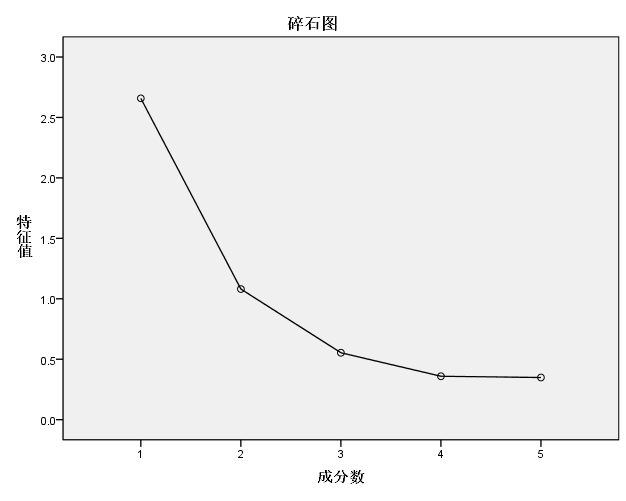


**Figure S3 Scree plot of the self-awareness scale**


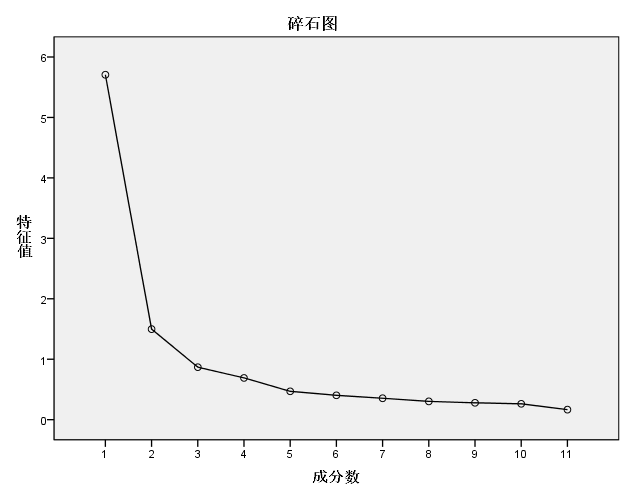


**Figure S4 Scree plot of the organizational support scale**
