# Supplementary material for: Psychometric properties of the Chinese version of the Perinatal Bereavement Care Confidence Scale (C-PBCCS) in nursing practice
Source: PLoS One. 2022 Jan 21;17(1):e0262965. doi: 10.1371/journal.pone.0262965 (PMC8782403; doi:10.1371/journal.pone.0262965)
Supplement: S4 File — (DOC) [file pone.0262965.s004.doc]

**Supplementary file 4 Rotated factor analysis of the C-PBCCS (n = 304)**

**Table S1 Rotated factor analysis of the bereavement support knowledge scale**

| **Bereavement support knowledge scale** | **Rotated factor analysis** | | |
| --- | --- | --- | --- |
| **Items** | **1** | **2** | **3** |
| a10 | 0.783 | 0.293 | -0.026 |
| a9 | 0.773 | -0.025 | -0.038 |
| a4 | 0.700 | 0.147 | 0.356 |
| a5 | 0.675 | 0.120 | 0.297 |
| a12 | 0.636 | 0.409 | -0.001 |
| a13 | 0.583 | 0.325 | 0.252 |
| a7 | 0.136 | 0.854 | -0.040 |
| a8 | 0.180 | 0.839 | 0.122 |
| a11 | 0.218 | 0.749 | -0.050 |
| a6 | 0.255 | -0.140 | 0.766 |
| a15 | 0.013 | -0.080 | 0.748 |
| a2 | -0.049 | 0.124 | 0.704 |
| a3 | 0.321 | 0.118 | 0.561 |

**Table S2 Rotated factor analysis of the bereavement support skills scale**

| **Bereavement support skills scale** | **Rotated factor analysis** | |
| --- | --- | --- |
| **Items** | **1** | **2** |
| b7 | 0.802 | 0.260 |
| b8 | 0.785 | 0.236 |
| b9 | 0.714 | 0.293 |
| b6 | 0.657 | 0.097 |
| b2 | -0.004 | 0.829 |
| b3 | 0.346 | 0.782 |
| b1 | 0.412 | 0.672 |
| b4 | 0.460 | 0.671 |

**Table S3 Rotated factor analysis of the self-awareness scale**

| **Self-awareness scale** | **Rotated factor analysis** | |
| --- | --- | --- |
| **Items** | **1** | **2** |
| c7 | 0.814 | 0.137 |
| c1 | 0.795 | 0.086 |
| c6 | 0.743 | 0.306 |
| c3 | 0.597 | 0.452 |
| c2 | 0.514 | 0.412 |
| c4 | 0.067 | 0.855 |
| c5 | 0.236 | 0.844 |
| c8 | 0.389 | 0.650 |

**Table S4 Rotated factor analysis of the organizational support scale**

| **Organizational support scale** | **Rotated factor analysis** | |
| --- | --- | --- |
| **Items** | **1** | **2** |
| d7 | 0.824 | 0.062 |
| d1 | 0.820 | 0.138 |
| d4 | 0.820 | 0.064 |
| d8 | 0.802 | 0.212 |
| d5 | 0.763 | 0.241 |
| d2 | 0.756 | 0.069 |
| d9 | 0.748 | 0.160 |
| d10 | 0.721 | 0.049 |
| d3 | 0.622 | 0.317 |
| d6 | 0.092 | 0.915 |
| d11 | 0.168 | 0.882 |
