# Supplementary material for: Psychometric properties of the Chinese version of the Perinatal Bereavement Care Confidence Scale (C-PBCCS) in nursing practice
Source: PLoS One. 2022 Jan 21;17(1):e0262965. doi: 10.1371/journal.pone.0262965 (PMC8782403; doi:10.1371/journal.pone.0262965)
Supplement: S5 File — (DOC) [file pone.0262965.s005.doc]

**Supplementary file 5 The results of factor analysis of the four scales of C-PBCCS**

| Items | Cronbach's α if  item deleted | Average mean | SD |
| --- | --- | --- | --- |
| Bereavement support knowledge scale (13 items)  丧亲护理知识量表 (Cronbach's α=0.835; Guttman Split-Half coefficient=0.878) |  | 43.09 | 6.66 |
| a2 Bereaved parents require the support of midwives to cope with their loss  父母需要助产士帮助他们应对围产期丧失 | 0.838 | 4.13 | 0.76 |
| a3 I understand that grieving is a process  我清楚丧亲父母的悲伤过程 | 0.824 | 3.80 | 0.88 |
| a4 I know how to provide the specific bereavement support needs of grieving mothers  我知道如何为悲伤的母亲提供针对性的帮助 | 0.808 | 3.37 | 0.86 |
| a5 I understand the cultural needs of bereaved parents  我知道不同文化背景/民族父母对丧亲护理的需求 | 0.816 | 3.36 | 0.95 |
| a6 I understand the social needs of bereaved parents  我知道丧亲父母的悲痛需要社会的关注和支持 | 0.832 | 4.03 | 0.75 |
| a7 I do not know the legal process associated with perinatal loss before 24 weeks gestation  我不知道孕周＜24周围产期丧失的有关法律制度 | 0.830 | 2.65 | 0.97 |
| a8 I do not know how to provide the specific bereavement support needs of grieving fathers  我不知道如何为悲伤的父亲提供针对性的帮助 | 0.820 | 2.88 | 0.96 |
| a9 I understand the religious needs of bereaved parents  我知道不同宗教背景丧亲父母的护理需求 | 0.826 | 3.15 | 0.92 |
| a10 I know the referral system for additional bereavement support  我了解可以为丧亲父母提供更多帮助的转诊体系 | 0.812 | 3.09 | 0.90 |
| a11 I do not have adequate practical knowledge for bereavement support  我缺乏丧亲支持的实践知识 | 0.826 | 2.46 | 0.94 |
| a12 I know the legal process associated with perinatal loss after 24 weeks gestation  我知道孕周＞24周围产期丧失的有关法律制度 | 0.813 | 2.92 | 0.95 |
| a13 I have been well prepared to provide perinatal bereavement support  我已经做好了提供围产期丧亲支持的准备 | 0.812 | 3.21 | 0.92 |
| a15 All maternity care professionals at the hospital should receive perinatal bereavement education  医院所有的产科医护人员都应接受围产期丧亲教育 | 0.837 | 4.03 | 0.72 |
| Bereavement support skills scale (8 items)  丧亲护理技能量表 (Cronbach's α=0.862; Guttman Split-Half coefficient=0.906) |  | 24.77 | 4.859 |
| b1 I have the skills to provide practical support to recently bereaved parents  我具备为近期经历丧亲事件的父母提供支持的能力 | 0.836 | 3.07 | 0.90 |
| b2 I do not have adequate perinatal bereavement support experience  我缺乏提供围产期丧亲支持的经验 | 0.865 | 2.47 | 0.90 |
| b3 I have grief counselling skills for providing psychological support to bereaved parents  我具备为丧亲父母提供心理支持的哀伤辅导技巧 | 0.836 | 2.96 | 0.84 |
| b4 I can provide the relevant information required by bereaved parents  我能够为丧亲父母提供他们需要的相关信息 | 0.831 | 3.06 | 0.84 |
| b6 I can provide emotional care to bereaved parents  我能够为丧亲父母提供情感关怀 | 0.866 | 3.85 | 0.73 |
| b7 I can provide spiritual care to bereaved parents  我能够为丧亲父母提供灵性照护（指为患者提供符合个体文化、信仰需求的照护） | 0.839 | 3.28 | 0.86 |
| b8 I can easily respond to the needs of bereaved sibling when accompanying their parents  我可以满足丧亲父母现存子女的情感需求 | 0.840 | 2.97 | 0.86 |
| b9 I can easily respond to the needs of bereaved parents expecting their next baby  我可以满足丧亲父母期望再次妊娠的特定需求 | 0.850 | 3.10 | 0.86 |
| Self awareness scale (8 items)  自我意识量表 (Cronbach's α=0.852; Guttman Split-Half coefficient=0.868) |  | 27.67 | 4.41 |
| c1 I am aware of the needs of recently bereaved parents  我清楚近期经历丧亲事件父母的需求 | 0.838 | 3.16 | 0.82 |
| c2 I can easily empathise with grieving parents (Empathy means that I can emotionally put myself in their place)  我能感同身受丧亲父母的悲伤 | 0.841 | 3.63 | 0.83 |
| c3 I am conscious of the particular needs of bereaved parents expecting their next baby  我清楚期望再次妊娠的丧亲父母的特定需求 | 0.828 | 3.43 | 0.77 |
| c4 I am aware of my limitations in relation to the provision of perinatal bereavement support  我知道自己在提供丧亲支持方面的不足之处 | 0.845 | 3.76 | 0.71 |
| c5 I am aware of my learning needs regarding bereavement support  我知道自己关于丧亲支持方面的学习需求 | 0.829 | 3.65 | 0.76 |
| c6 I am regularly engaged in reflective practice in relation to the provision of perinatal bereavement support  我经常对丧亲支持的实践经历进行反思 | 0.828 | 3.19 | 0.83 |
| c7 I am aware of my personal resources for bereavement support  我清楚自己提供丧亲支持所具备的个人资源（能力、知识等） | 0.828 | 3.20 | 0.83 |
| c8 Being aware of my need for support in relation to providing care for bereaved parents encourages me to seek help  我意识到在照护丧亲父母方面需要支援，这促使我寻求帮助 | 0.833 | 3.66 | 0.73 |
| Organizational support scale (11 items)  组织支持量表 (Cronbach's α=0.901; Guttman Split-Half coefficient=0.933) |  | 33.74 | 6.60 |
| d1 I have support from my workplace management in relation to providing bereavement support  我得到了医院管理层在提供丧亲支持方面的帮助 | 0.885 | 3.15 | 0.87 |
| d2 I have adequate peer support in my work place in relation to providing bereavement support  我得到了医院同事在提供丧亲支持方面的帮助 | 0.890 | 3.23 | 0.84 |
| d3 My work environment allows me to feel relaxed to carry out my daily work  我的工作环境让我轻松地完成每天工作 | 0.894 | 3.24 | 0.85 |
| d4 I get recognition for providing effective bereavement support  我因为提供了有效的丧亲支持而得到认可 | 0.890 | 3.18 | 0.81 |
| d5 The manager organises my daily work placements to facilitate me to provide bereavement support  领导对我日常工作内容的安排，有利于我提供丧亲支持 | 0.887 | 3.13 | 0.80 |
| d6 My workload hinders effective bereavement support.  我的工作量阻碍了我提供有效的丧亲支持 | 0.908 | 3.03 | 0.88 |
| d7 There is a clear policy in my ward/unit for the provision of bereavement support to parents  我所在的科室有丧亲支持相关的明确政策 | 0.888 | 2.85 | 0.82 |
| d8 There is adequate number of midwives to cover the ward/unit to enable the provision of bereavement support.  有足够的护士/助产士来满足科室丧亲支持的提供 | 0.885 | 2.99 | 0.86 |
| d9 My organisation provides bereavement support training  我所在的医院提供丧亲支持培训 | 0.889 | 2.88 | 0.85 |
| d10 Debriefing opportunities are always provided for me when required following a traumatic incident.  在经历围产期丧亲的创伤事件后，我总是有机会进行病例分享和交流 | 0.891 | 3.04 | 0.84 |
| d11 The workload of the ward/unit hinders effective bereavement support  科室的工作量阻碍了有效丧亲支持的提供 | 0.906 | 3.04 | 0.88 |
