# Supplementary material for: Psychometric properties of the Chinese version of the Perinatal Bereavement Care Confidence Scale (C-PBCCS) in nursing practice
Source: PLoS One. 2022 Jan 21;17(1):e0262965. doi: 10.1371/journal.pone.0262965 (PMC8782403; doi:10.1371/journal.pone.0262965)
Supplement: Attachment — Submitted filename: Response to Reviewers.docx [file pone.0262965.s009.docx]

**Author response**

**Response to academic editor**

**Specific comments:**

**Comment 1:**

*In Table 1, personality of the participants was reported. How to categorise the participants into three different personalities? Which instrument was adopted for assessment? What's the significance of this data to the results?*

**Reply:**

Thank you for the editor’s valuable comment. We did not use an instrument to assess participants’ personality. Participants chose personality according to their subjective judgements. Given that participants’ personality has little relation with the results in our study, we have removed it from Table 1 to avoid ambiguity. Please see Table 1.

**Comment 2:**

*Some grammatical errors which affect the clarity, especially the revised and newly added parts.*

**Reply:**

Thank you for the editor’s careful reviewing. We have invited two native speakers to polish the revised manuscript. Some grammatical errors have been corrected.
